# Adenocarcinoma arising in an ectopic enterogenous cyst: A rare case report and review of literature

**DOI:** 10.3389/fonc.2022.942449

**Published:** 2022-12-06

**Authors:** Haina Du, Dachao Xu, Shuhui Zhang, Xinliang Zhang, Mingzhi Fang, Min Li

**Affiliations:** ^1^ Department of Oncology, Nanjing Hospital of Chinese Medicine Affiliated to Nanjing University of Chinese Medicine, Nanjing, China; ^2^ Department of Anorectal, Nanjing Hospital of Chinese Medicine Affiliated to Nanjing University of Chinese Medicine, Nanjing, China

**Keywords:** enterogenous cyst, malignant transformation, anterior sacral, soft tissue, adenocarcinoma

## Abstract

Enterogenous cyst (EC) is a rare congenital lesion generally located in the central nervous system, such as in the cerebral hemispheres, posterior fossa, or spinal canal. They are usually benign lesions, and malignant transformation is rare. A 42-year-old woman felt an obvious pain in the lump and went to a local hospital for local lumpectomy. After 7 months, she again felt pain in the buttocks and difficulty in urinating and defecation. The computed tomography (CT) scan showed a mass in the pelvis. Sacrococcygeal cyst excision was performed 10 days later, and postoperative pathology showed epidermoid cyst. Shortly after, the patient recovered and was discharged from the hospital; the pain in the buttocks continued to recur. Puncture and drainage were performed five times. Later, the patient went to our hospital for treatment, and pelvic MRI showed multiple abnormal signal shadows in the presacral and sacrococcygeal regions, some of which were considered abscesses, and some were cystic lesions. She underwent tumor resection and was diagnosed with EC with locally moderately differentiated adenocarcinoma. Four months later, the patient’s symptoms of swelling and pain recurred. MRI examination showed multiple high-signal T2 shadows in the anterior sacral and subcutaneous tissues of the buttocks, and enhanced scan showed partial marginal enhancement. After assessment, the patient was given a radiation dose of 60 Gy/25F. ECs in the anterior sacral and soft tissue of the buttocks are very rare, and the case of carcinomatous transformation has never been reported. Therefore, we discussed the clinicopathological features of ectopic ECs and reviewed the literature.

## Introduction

Enterogenous cyst (EC) is considered as a rare disorder of dermal development. EC occurs during the third week of intrauterine life and evolves from the remnant or ectopic tissue of the neural tube and the gastrum (1). The tumor is mostly located in the spinal canal of the cervical and thoracic vertebrae of the central nervous system and is often accompanied by abnormal vertebral body morphology and function. In addition, ECs occurring in the mediastinum, abdominal cavity, and intracranium have also been reported. However, cyst location in the anterior sacral and soft tissue is very rare, and concurrent malignant transformation of cysts has never been reported.

We reported a case of EC with adenocarcinoma transformation in presacral and subcutaneous soft tissues and reviewed relevant literature to collect its clinical characteristics, providing reference for the diagnosis and treatment of such patients.

## Case presentation

### Clinical presentation

A 42-year-old woman revealed that, during childhood, a soft tissue mass near the anus was found. The mass was about 4 cm × 5 cm in size, with a slightly tough texture, and she did not experience pain; thus, she received no special attention. In 2019, she felt an obvious pain in the lump and went to the hospital for local lumpectomy. After surgery, a deep sinus was found, and the doctor repeatedly irrigated the lump with normal saline. After 7 months, she again felt pain in the buttocks and experienced difficulty in urination and defecation. She was readmitted to the hospital, and the computed tomography (CT) scan showed a mass in the pelvis. Vaginal puncture and drainage guided by B ultrasound was performed to relieve difficulty in defecation. Sacrococcygeal cyst excision was performed 10 days later, and postoperative pathology showed epidermoid cyst. The pain in the buttocks continued to recur after the patient was discharged from the hospital. After that, puncture and drainage were performed five times under the guidance of B ultrasound. The last time a drainage tube was placed in the tumor, about 25 ml of yellowish clear liquid was drawn out every day. Later, the patient went to our hospital for treatment.

### Imaging and laboratory features

**Figure 1:**
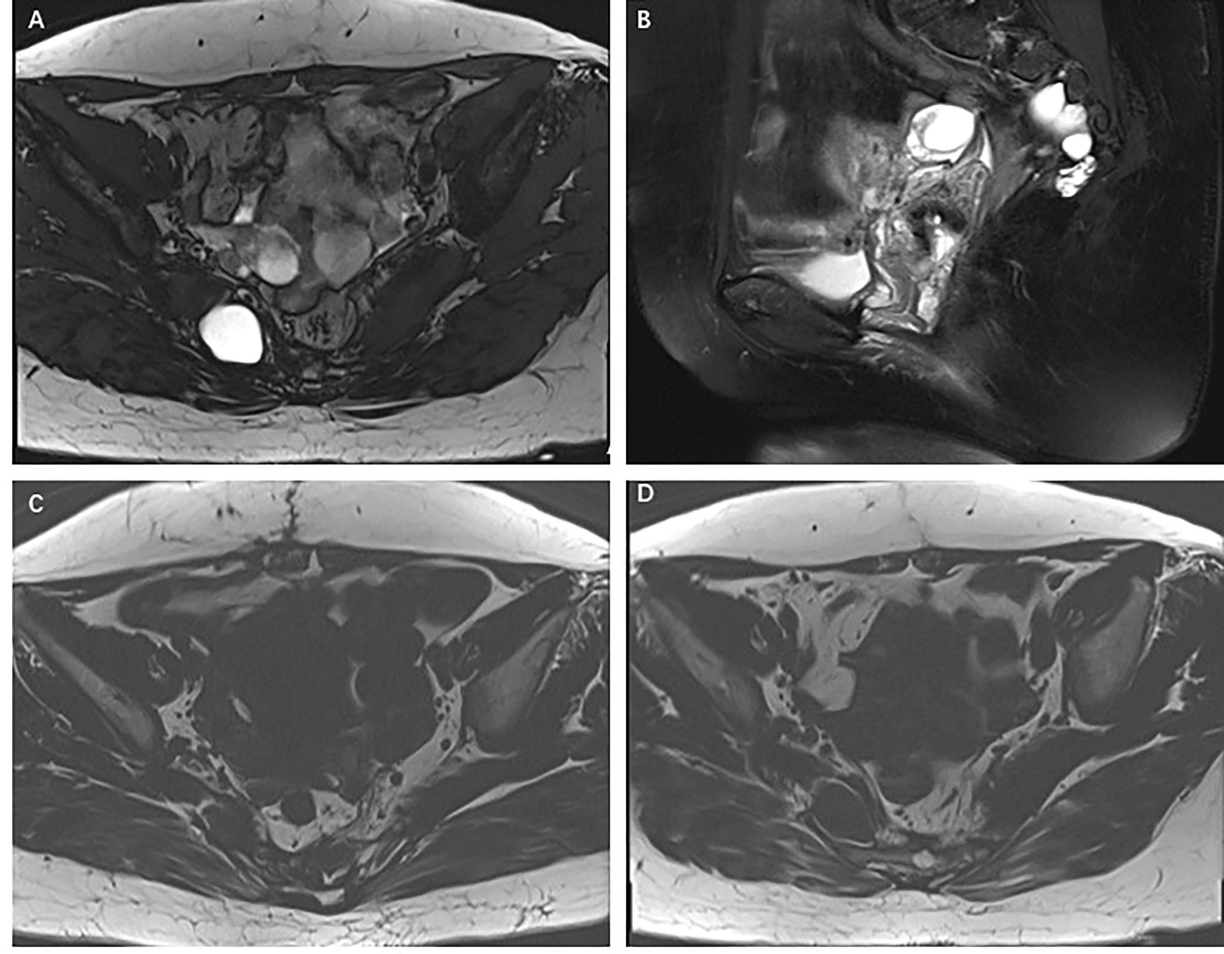
**(A–C)** T1- and T2-weighted MRI scans demonstrating multiple ventricular T2 hypersignal and T1 hypointense cystic lesions in the right buttock tissue and sacrococcygeal region. **(D)** T2-weighted gadolinium-enhanced MRI scans demonstrating annular enhancement of the cystic wall, but no enhancement of the cystic part of the lesion.

**Figure 2:**
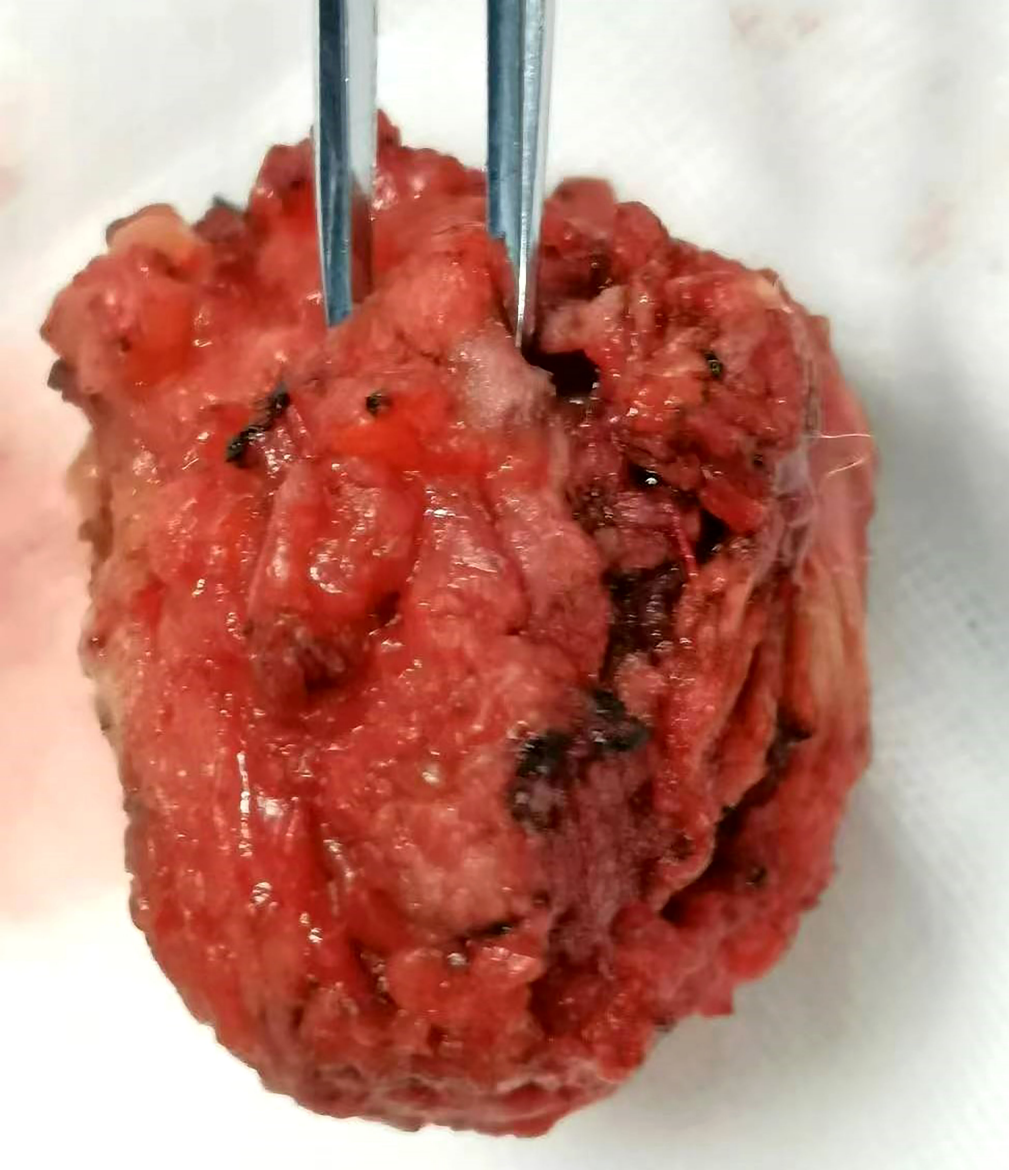
Resected lump specimens (4 cm × 3 cm).

Pelvic magnetic resonance imaging (MRI) showed multiple abnormal signal shadows in the presacral and sacrococcygeal regions, some of which were considered abscesses. MRI scans showed a multiloculated T1 hypointense and T2 hyperintense cystic lesion in the right buttock tissue and sacrococcygeal region. Annular enhancement of the cystic wall was observed on the T2-weighted gadolinium-enhanced MRI scans (**Figure 1**). Colonoscopy showed no obvious abnormality. The admission laboratory examination showed that the levels of tumor serum markers, such as CEA, CA199, AFP, and CA724, were the scope of the standard. Also, there were no signs of infection. Surgical resection was performed again, and a mass of about 4 cm × 3 cm was removed (**Figure 2**). Unfortunately, the tumor could not be completely removed.

### Pathological and immunohistochemical characteristics

**Figure 3:**
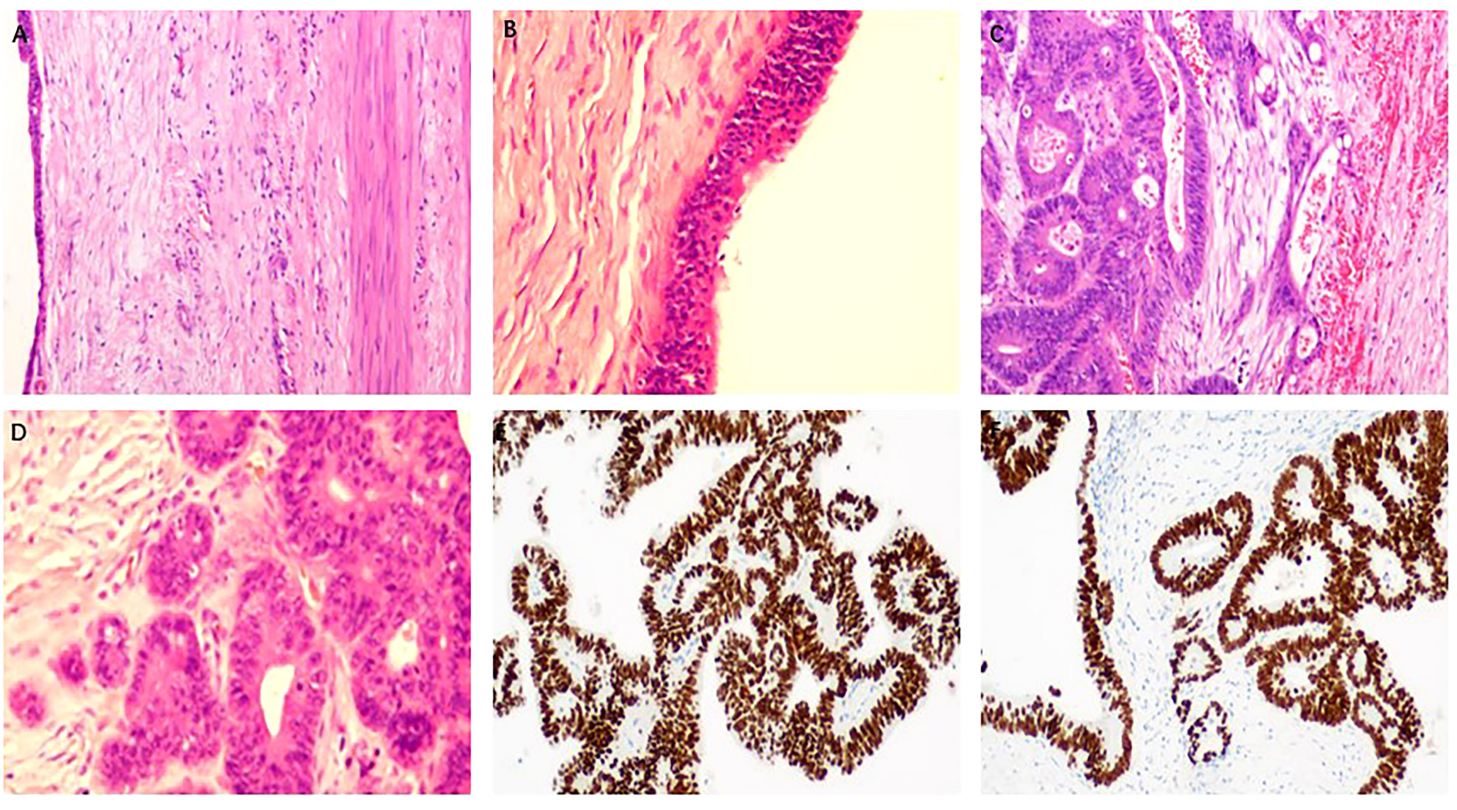
Histopathological features of malignant transformation of EC. **(A, B)** Cuboid columnar epithelium on smooth muscle tissue of cyst wall. **(C)** Focal cyst wall shows adenocarcinoma with interstitial invasion, and cancer tissue breaks through the basement membrane and invades the interstitium. **(D)** In the lesion area, glands of different sizes and disorderly arrangement can be seen, and the cancer cells are irregular in shape, different in size, and different in nuclear staining. **(E, F)** Immunostaining indicated positivity for CDX2 **(E)** and SATB2 **(F)** (original magnification: **(B, D)** ×200; **(A, C, E, F)** ×100).

The final histological diagnosis was EC with locally moderately differentiated adenocarcinoma (**Figure 3**). Outside the cyst wall is fibrous connective tissue and smooth muscle (**Figures 3A, B**). The specimen showed benign cyst lesions covered by a single layer of columnar or cuboidal epithelium. Some of the cyst walls were lined by dysplastic epithelium, with interstitial infiltration. Local cancerous lesions showed glands of different sizes and disorderly arrangement (**Figures 3C, D**). Immunohistochemical staining of SATB2 and CDX2 in the cyst epithelial cells showed strong nuclear staining (**Figures 2E, F**). In addition, MLH1, MSH2, MSH6, PMS2, CDH-17, and CK20 and other specific molecules were also positive, and CK7 was negative. All the above immunohistochemical results showed the same immune characteristics as intestinal adenocarcinoma.

### Treatment options and follow-up

Four months later, the patient’s symptoms of swelling and pain recurred. Considering the recurrence of the lesion, the patient was given a radiation dose of 60 Gy/25F. For the rare disease, there is insufficient evidence on the role of adjuvant chemotherapy. Given the immunohistochemical results, a fluorinated pyrimidine- and oxaliplatin-containing regimen was recommended, but the patient declined chemotherapy. We plan to identify KRAS, NRAS, and BRAF mutations and evaluate MSI status to guide subsequent individualized therapeutic and oncological prognosis for the patient, which failed due to the patient’s financial reasons. As of 20 July 2022, the patient had a good outcome, and a recently repeated MRI scan did not show any evidence of cyst recurrence. This indicates that radiotherapy shows a good local control effect.

## Discussion

ECs are considered congenital abnormalities. The etiology and pathogenesis of ECs are not clear. Most scholars believe that the ectoderm and endoderm are tight during the early embryonic period. At the third week of the embryo, they separated with the development of the embryo (the ectoderm developed neural tube, the endoderm differentiated into intestinal tube), and the middle was only linked by the nerve-intestinal sac zone. If the embryo development separation disorder, residual or ectopic, it will form perineural EC, often combined with gastrointestinal, spinal, spinal cord and other malformations (2).

ECs mostly occur in the central axis, with cervical segment and upper thoracic segment being more common, lumbosacral department being rare, and other parts being rarer (3). The clinical symptoms of ECs are not typical and are often related to the site of occurrence, mainly radiculopathy and spinal cord compression at the corresponding site of the lesion. Most patients may be associated with congenital spinal malformations, such as vertebral fusion, hemivertebra, spina bifida, and butterfly vertebra. The main manifestations of intracranial ECs are intermittent headache and dizziness, which may be followed by seizures, cerebral nerve palsy symptoms, limb movement disorders, and increased intracranial pressure symptoms.

The occurrence of ECs in the anterior sacral and hip soft tissue is very rare, and ECs with malignant transformation are rarely reported. We reported a case of sacral caudal EC with carcinogenesis. After a PubMed search, we retrieved a total of nine literatures in **Table 1**, which had two parts. One part involved the cases with similar lesion sites to our case; only one case was located in the anterior sacral area, one case was located in soft tissue, and none of them showed signs of malignant transformation (4, 5). ECs occurring in the sacral and soft tissue are very rare. Li et al.’s surgical analysis of 33 patients with presacral tumors discovered that one patient had benign EC with abdominal mass as the initial symptom (5). Mantoo et al. reported an EC in the subcutaneous tissue of scapula with pain and a growing mass, which lasted several years. EC was diagnosed after surgery and there was no recurrence after surgery (4). Our case occurred in the subcutaneous tissue of the buttocks, with similar symptoms, such as swelling and pain. Multiple punctures were performed to discharge pus and relieve pressure. In addition, our lesions also occurred in the sacral anterior area, resulting in the patient experiencing difficulty in defecation.

**Table 1:** Characteristics of cases with ECs from published literature.

	Type								
	Similar lesion site		Malignant transformation						
**Author**	Mantoo et al. (4)	Li et al. (5)	Jeffrey (6)	Ho et al. (7)	Sahara et al. (8)	Monaco et al. (9)	Hill et al. (10)	Gessi et al. (11)	Tsutsumi et al. (12)
**Site**	Scapula soft tissue	Anterior sacral	Retroperitoneal	Right hemisphere	Foramen magnum	Posterior fossa	Retroperitoneal	Posterior fossa	Cervical spine
**Published year**	2008	2011	2007	1998	2001	2003	2004	2008	2020
**Sex**	M	M	M	F	M	M	F	M	M
**Age**	46	63	54	45	53	36	33	25	43
**Nation**	Singapore	China	UK	USA	Japan	Italy	Australia	Italy	Japan
**First symptom**	Pain Increasing lump	Abdominal mass	Heartburn	Abnormal sensation	Neck pain	Headaches Vomiting Drowsiness	Left loin pain	Hypoacusis	Numbness of right limb
**Duration**	Several years	2 months	4 years	NA	NA	Several months	NA	6 years	1 month
**Elevated tumor markers**	NA	NA	CA199	None	NA	NA	NA	NA	CA199
**Therapy**	S	S	S	S	S+R	S	S	S	S
**Cancerization**	No	No	Yes	Yes	Yes	Yes	Yes	Yes	Yes
**Pathological type**			Adenocarcinoma	Adenocarcinoma	Adenocarcinoma	Intraepithelial carcinoma	Adenocarcinoma	Adenocarcinoma	Adenocarcinoma
**Recurrence**	No evidence	No evidence	Yes	Yes	Yes	No	Yes	Yes	Yes
**Follow-up**	6 months	29 months	1 month (dead)	24 months	42 months (dead)	24 months	NA	6 months (dead)	6 months (dead)

M, male; F, female; (dead), died of the disease; S, surgery; S+R, surgery + radiotherapy; NA, Not Applicable.

The other part included seven cases of ECs undergoing malignant transformation from the central nervous system and retroperitoneum (6–12), which were not collected and discussed before. Among the seven cases of malignant transformation of ECs, the pathological type of the cases reported by Monaco et al. was intraepithelial carcinoma, and the other four cases developed adenocarcinoma and recurred. We also found that only two cases reported elevated serum tumor markers, both of which were CA199 (6, 12). It is speculated that there are no specific serum tumor markers for malignant ECs.

In our current case, several aspects are worth discussing. First, the site of occurrence is relatively special, occurring in the anterior sacrum and subcutaneous tissue of the buttocks, which we ascribed to ectopic ECs. For ectopic ECs, some scholars also named them isolated ECs, that is, cysts occurring outside the gastrointestinal tract with no direct anatomical connection and common blood supply with adjacent tissues and pathological features of gastrointestinal wall (13). The mechanism of ECs in the sacral caudal and subcutaneous tissues is unclear. Mantoo et al. speculated that the migration of cells from the foregut during intrauterine development plays an important role in the process of occurrence (4). Specifically, during the first 3 weeks of embryonic development, due to incomplete separation of mesoderm and endoderm, the unseparated endoderm tissue develops into the foregut, which then forms ECs. In addition, we hypothesized that ectopic implantation of intestinal epithelium caused by injury may be one of the possible causes of isolated ECs. In the cases of the ectopic cyst we mentioned above (4, 5), benign ECs occurring in the sacral presacral and soft tissue are characterized by a continuously growing mass, which can be cured by surgery and is not likely to recur. In our case, repeated recurrence after surgery may also be related to specific sites. Second, most of the ECs are benign and malignant transformation is rarely reported in the literature, especially in rare sites, such as the sacral caudal and subcutaneous soft tissue (14, 15). The exact molecular mechanism of malignant transformation remains unknown. A possible mechanism is that chronic inflammation is prone to malignant transformation due to repeated cyst rupture or subtotal resection of the cyst wall. Alternatively, long-term carcinoma *in situ* lesions may lead to malignant transformation (11, 16). We first report a case of ectopic EC showing malignant transformation of moderately differentiated adenocarcinoma.

Clinical and radiological examinations will likely fail in identifying malignant ECs, which can be confirmed by pathological and immunohistochemical staining. The epithelial cells forming the wall of NC cysts can be flat, cubic, columnar, pseudo-laminated columnar, laminated squamous epithelium, etc. In some cases, more than two epithelial components may be seen, with intermigration. The outer layer of the cyst wall is composed of fibrous connective tissue, and melanin deposition, cartilage, smooth muscle, mucus gland, fat, and calcification can be seen in some cases. Our immunohistochemical staining results show that intestinal epithelial-specific transcription factors CDX-2, cadherin CDH-17, special AT-rich sequence-binding protein SATB-2, and cytokeratin 20 (CK20) were all positive, while CK7 was negative, suggesting a foregut origin. CDX2 is a gut-specific nuclear transcription factor and a key regulatory protein for intestinal epithelial formation and differentiation. In gastrointestinal tumors, it is mainly expressed in the small intestine and colorectum, which can be used as a differential indicator of the primary site of tumors. CK20 and the Merkel cell-derived marker of gastrointestinal epithelial transitional epithelium, used in gastrointestinal adenocarcinoma, are often positive for bowel cancer. CK7 (cytokeratin 7), a marker of epithelial origin, is usually expressed in adenocarcinomas, expressed in glandular and transitional epithelial cells, and not expressed in cells of non-epithelial origin. Adenocarcinoma of the gastrointestinal tract was negative. SATB2 is strongly expressed in adult lower gastrointestinal tract and colorectal epithelial cells. SATB2 has a good positive rate in colorectal cancer, and its sensitivity and specificity are high. CK7–/CK20+ carcinomas make up over 90% of cases of colorectal carcinomas (17).

Surgery is the preferred treatment and it is widely believed the cyst should be removed regardless of the symptoms. The key to the operation is whether the capsule wall can be completely removed; otherwise, it will relapse and regenerate due to residual lesions. Once recurrence occurs, reoperation may be considered. In our case, due to the difficulty of complete resection and the malignant transformation of the lesion, postoperative local radiotherapy was combined with residual lesion. Through literature review, we found that only one patient with a tumor located in the foramen magnum received radiotherapy (50 Gy) and chemotherapy (including carboplatin and etoposide), but died 1 year after the second surgery (8). In addition, Jeffrey reported a case of a retroperitoneal EC with adenocarcinoma *in situ*, in which fluoropyrimidine and oxaliplatin were planned for the intestinal tumor, but the patient died of metastatic disease before chemotherapy (6). In the other cases, only surgical resection was performed (7, 9–12). For the treatment of malignant ECs, the choice of postoperative chemotherapy and radiotherapy is worthy of further investigation.

Since ectopic ECs are rarely reported and easily misdiagnosed as other diseases, more cases are needed to collect and summarize their characteristics. Most cases reviewed in this paper are from case reports, and the standards are not uniform, leading to limitations in our conclusions. Further observation and comparative studies are needed.

## Data availability statement

The original contributions presented in the study are included in the article/supplementary material. Further inquiries can be directed to the corresponding author.

## Ethics statement

Written informed consent was obtained from the individual(s) for the publication of any potentially identifiable images or data included in this article.

## Author contributions

HD and DX are the common first authors and the major contributors in writing the manuscript. SZ and ML edited the manuscript and approved the final version. XZ and MF were responsible for reviewing the literature and collecting the information of the patients. All authors listed have made a substantial, direct, and intellectual contribution to the work and approved it for publication.
